# Impact of COVID-19 on the Volume of Pediatric Surgery Cases in a Referral Hospital in the Al-Madina Region of Saudi Arabia

**DOI:** 10.7759/cureus.50123

**Published:** 2023-12-07

**Authors:** Muayad A Alfarsi, Najwa A Alqurashi, Raneem O Alahmadi, Rawan T Alhejaili, Dana M Ismaeel, Aisha A Jamal

**Affiliations:** 1 General Surgery/Pediatric Surgery, Taibah University, Madinah, SAU; 2 College of Medicine, Taibah University, Madinah, SAU

**Keywords:** impact, pediatric surgery, pandemic, health care, covid-19

## Abstract

Background

The COVID-19 pandemic has affected healthcare systems worldwide, leading to prioritizing resources for the diagnosis and management of COVID-19 cases. As a result, providing medical care to patients with health conditions, including pediatric surgeries, has been significantly affected.

Objectives

The objective of this research is to evaluate the impact of the COVID-19 pandemic on the prevalence of pediatric surgeries in a referral hospital in Al-Madina, KSA.

Method

An observational retrospective cross-sectional study was conducted in a referral hospital in the Al-Madina region of Saudi Arabia. We retrospectively reviewed all pediatric surgical records that met the inclusion criteria from March 2018 to March 2022.

Results

Out of the patients who underwent surgeries (5704), it was indicated that the number of patients who underwent surgeries during the COVID-19 pandemic (42.7%) was less than those who underwent surgeries before the COVID-19 outbreak (57.6%). The number of patients presented to the OPD significantly increased during the COVID-19 period compared to before COVID-19. The number of emergency cases before and during COVID-19 was similar. The period between diagnosis and surgical intervention for emergency cases was significantly shortened during the COVID-19 pandemic. The hospital stay period significantly decreased during the pandemic.

Conclusion

The study found that the pandemic has caused a considerable decline in the number of pediatric surgery cases compared to the previous years. The longer observation period of this study likely allowed for a better understanding of the pandemic situation, which contributes to strengthening our understanding of the pandemic's impact on healthcare services.

## Introduction

A new coronavirus belonging to the SARS-CoV-2 family was discovered in Wuhan, China, in December 2019. The World Health Organization (WHO) declared the disease a pandemic on March 11, 2020 [1]. On March 2, 2020, the Saudi government declared the first case of coronavirus disease 2019 (COVID-19). The cumulative number of cases by April 2023 in Saudi Arabia reached 841,469 cases, with the Al-Madina region being the fifth most prevalent, with 37,294 cases [2].

COVID-19 can lead to pneumonia of varying degrees and acute respiratory distress syndrome, and, in severe cases, may require ventilation or intensive care unit admission [3]. Global efforts have been united during the pandemic to serve, educate, and train healthcare providers (HCPs) in order to prepare and support the workforce and resources required to confront the pandemic [4].

Having prior experience with MERS-CoV, the KSA has established the basis and methods for combating the COVID-19 pandemic. In response to the pandemic, the Ministry of Health (MOH) of Saudi Arabia has taken effective preventive measures and prepared hospitals to contain a large number of patients, setting an example for other regions and countries to follow [5].

The COVID-19 pandemic has affected healthcare systems worldwide, going far beyond infected people, leading to the prioritization of resources for the diagnosis and management of COVID-19 cases. As a result, providing medical care to patients with other health conditions, including pediatric surgeries, has been significantly affected. In order to deal with the pandemic, healthcare systems and organizations have made significant changes to routine services, which include deferring scheduled treatment and a partial shift to virtual care [6].

Moreover, concerns about infection risks and a desire not to overburden the system as it is managing the cases of COVID-19 caused a significant shift in patients' and parents’ healthcare-seeking behavior [6]. In a pan-European survey among surgical departments, most participants reported a high impact of the pandemic on surgical therapy, i.e., suspended surgical procedures, restriction of capacities, and a decrease in patient referrals [7].

Many studies have shown that a number of elective surgical cases were suspended [8-10], which may result in treatment delays and thus present as emergencies. A systemic review of 39 studies conducted across 19 countries found that all of the studies included showed a decrease in daily admissions to the pediatric emergency department (ED) during the period of social distancing measures [11].

The impact of the COVID-19 pandemic on pediatric surgeries in Al-Madina, Kingdom of Saudi Arabia (KSA), remains unclear, and there is a need to investigate the prevalence of pediatric surgeries during the pandemic. However, previous research on the epidemiology of pediatric surgery cases in Al-Madina is lacking, making it challenging to assess the change as no defined baseline is present. Further data are needed to help address the surgical burden on the healthcare system in Al-Madina. The goal of this research is to evaluate the impact of the COVID-19 pandemic on the prevalence of pediatric surgeries in a referral hospital in Al-Madina, KSA, by comparing the number of pediatric surgeries performed before and during the pandemic. The study aims to provide valuable insights into the impact of the pandemic on pediatric surgery as well as help predict future surgical needs and resource allocation. Furthermore, the results of this study will contribute to the development of strategies to ensure the continued provision of safe and effective care for children requiring pediatric surgery during future pandemics or other critical situations.

Research objectives

The research objectives of this study are to determine the general characteristics of the pediatric surgery population in the Al-Madina region and the effect of the COVID-19 pandemic on them, to investigate the influence of the pandemic on the volume of pediatric surgeries, and to compare pediatric surgery practices before and during the COVID-19 pandemic in our institution, to assess the impact of COVID-19 on providing timely, efficient care for emergency cases, and to evaluate the influence of COVID-19 on the outcomes of pediatric surgeries regarding hospital stay, complications, readmission, and reoperation.

## Materials and methods

Study design

This is an observational retrospective cohort study conducted in King Salman bin Abdulaziz Medical City (KSAMC), Maternity and Children Hospital (MCH), Al-Madina, Saudi Arabia, in the period from November 2021 to October 2022.

Study participants

They were recruited according to the inclusion criteria: children under the age of 14 years old, males and females with surgical conditions who either underwent surgery or were scheduled for surgery. All children over the age of 14 and all non-general pediatric surgery cases (neurosurgery, orthopedics, ENT, plastic surgeries, etc.) were excluded.

Sample size

**Figure 1:**
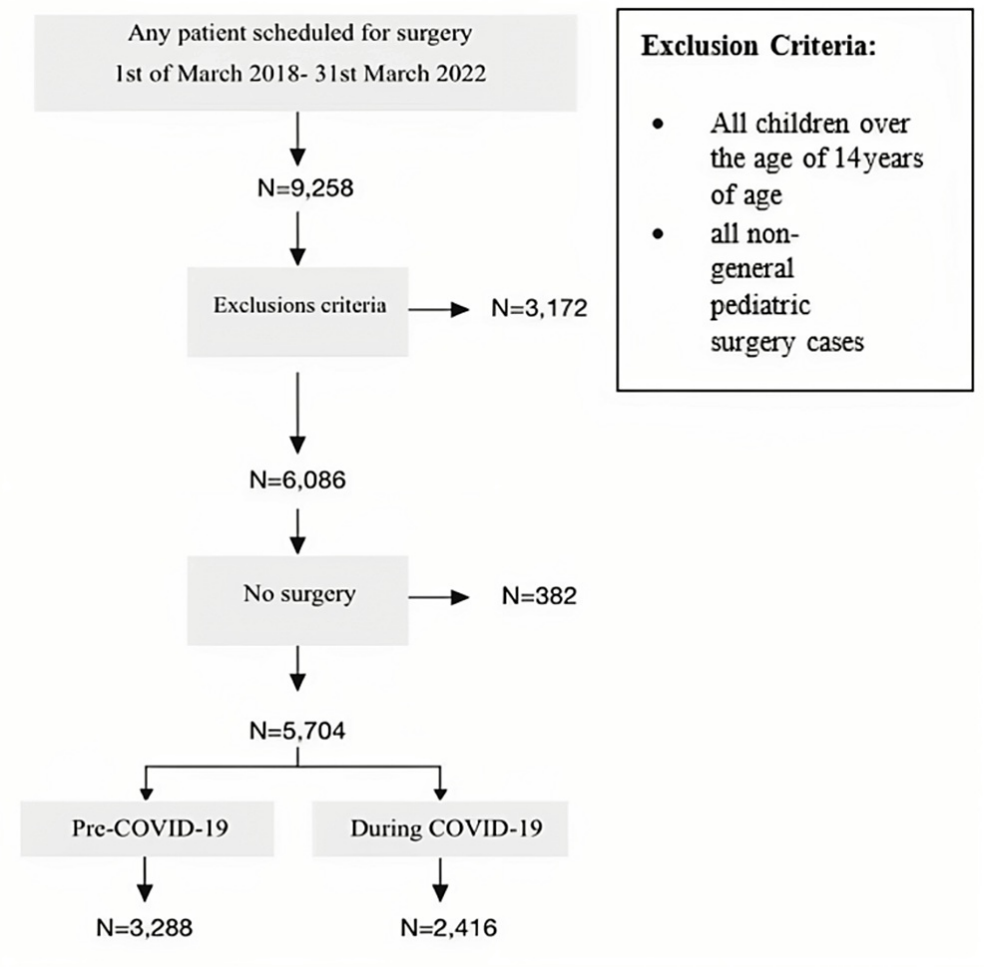
Patient flow chart and inclusion and exclusion criteria. Patients were stratified after being scheduled for surgery (pre-COVID-19 and during COVID-19) N: Number of cases

The sample size was based on the number of eligible patients who had general pediatric surgeries during the study periods; as such, no formal sample size calculation was conducted. The sample included all pediatric surgical records that met the inclusion criteria from March 2018 to March 2022; the data were categorized into control and variable groups. The pre-COVID-19 period from March 2018 to March 2020 represented the control group, while data from March 2020 to March 2021 represented the variable group. Details are described in Figure 1.

Data collection

Data were collected from medical records. The electronic records of all included children (1-14 years) admitted for surgery during the mentioned assigned date were retrospectively reviewed. For each admission, we recorded the medical record number, sex, age, date of birth, area of residence, date of presentation, venue of presentation (ED, outpatient department (OPD), ward, virtual clinic, neonatal intensive care unit (NICU), pediatric intensive care unit (PICU)), type of admission (elective, emergency, urgent), chief complaint, diagnostic investigations, principal and secondary diagnosis, procedures performed, duration from a time of diagnosis (based on the time of the diagnostic investigations) to a time of surgical intervention in hours calculated only for the cases presented to the ED. Additionally, we recorded the outcomes: hospital stay in days (calculated from the time of admission to the time of discharge), complications (defined as deviation from the normal postoperative course, which is determined based on operative notes, progress notes, nurse assessment notes, and investigations), if there was any readmission (based on hospital visits, including the date and the procedure that had been done), and reoperation. During data collection, it was found that there was missing data in some of the electronic files. To address this issue, some of the missing data was retrieved from the hard copies of these files. The data were filled out on an Excel sheet and kept confidential by the participant researchers.

Statistical analysis

After data extraction, it was revised and coded. Statistical calculations were done using IBM SPSS Statistics for Windows, Version 26 (Released 2019; IBM Corp., Armonk, New York, United States). Data were statistically described in the median (IQR) for continuous data. Frequencies (number of cases) and valid percentages were used for categorical variables. The Chi-square or Fisher’s exact test was used for categorical variables between the subgroups. The Mann-Whitney U test was used for continuous data. P-values less than 0.05 were considered statistically significant.

Ethical considerations

The research ethics committee of Taibah University's College of Medicine reviewed this work and gave their approval (Approval ID: STU-21-018). Anonymity for all participants in this study was considered. All data were stored on a private USB stick and were kept with the corresponding author.

## Results

Data from 6,086 patients were collected; a total of 5704 patients who underwent surgeries were included in the study. Data from 382 patients who did not experience any type of surgery were excluded. Out of them, 316 (82.7%) were canceled before COVID-19. Most of the patients were males (73.5%), young children (from one to five years old) (44.4%), underwent elective surgery (71%), and were admitted to the hospital with abdomen disorders (80.4%). All details are described in Table 1. By exploring the impact of the COVID-19 pandemic on the characteristics of patients, the COVID-19 pandemic had a significant effect on gender, age, and the venue of the presentation for those who underwent surgeries (p-value <0.001). The number of female patients significantly decreased during COVID-19 (24%) compared to before the COVID-19 period (28.4%) (p-value <0.001). In addition, the number of young children and children of school age who underwent surgeries significantly decreased during COVID-19 (43.3% and 26.4%, respectively) compared to the period before COVID-19 (45.2% and 31.5%, respectively) (p-value <0.001). Regarding the venue of the presentation, the number of patients presented to the OPD significantly increased during the COVID-19 period (66.1%) compared to before COVID-19 (61.3%) (p-value <0.001). Regarding surgery interventions, the repair of hernia was the most common type of intervention performed among the included patients (39.4%). However, during COVID-19, the frequency of this surgery significantly decreased compared to before COVID-19 (30.8% vs. 45.6%, p-value <0.001). All details are in Table 1.

**Table 1:** General characteristics of the study subjects N: Number of cases, ER: emergency room, NICU: neonatal intensive care unit, OPD: outpatient department, PICU: pediatric intensive care unit

Factors		Presentation Time		Total (n=5704)	p-value
	Category	Pre-COVID-19	During COVID-19		
Gender	Female	933 (28.4)	580 (24)	1513 (26.5)	<0.001
	Male	2355 (71.6)	1836 (76)	4191 (73.5)	
Age	Neonates/Infants (<1 year)	765 (23.3)	738 (30.5)	1503 (26.3)	<0.001
	Young Child (1-5 years)	1487 (45.2)	1039 (43)	2526 (44.3)	
	School Age (>5 years)	1036 (31.5)	639 (26.4)	1675 (29.4)	
Type of case	Elective	2327 (70.8)	1725 (71.4)	4052 (71)	0.871
	Emergent	952 (29)	685 (28.4)	1637 (28.7)	
	Urgent	9 (0.3)	6 (0.2)	15 (0.3)	
Venue of presentation	ER	761 (23.1)	580 (24)	1341 (23.5)	<0.001
	Neonatal intensive care (NICU)	119 (3.6)	98 (4.1)	217 (3.8)	
	Outpatient department (OPD)	2015 (61.3)	1597 (66.1)	3612 (63.3)	
	Pediatric Intensive Care Unit (PICU) /Virtual Clinic	9 (0.3)	19 (0.8)	28 (0.5)	
	Ward	384 (11.7)	122 (5.1)	506 (8.9)	
Surgical interventions (n= 2672)	Circumcision	440 (23.8)	457 (41)	897 (33.6)	<0.001
	Orchidopexy	407 (26.1)	315 (28.3)	722 (27)	
	Repair of hernia	710 (45.6)	343 (30.8)	1053 (39.4)	

**Figure 2:**
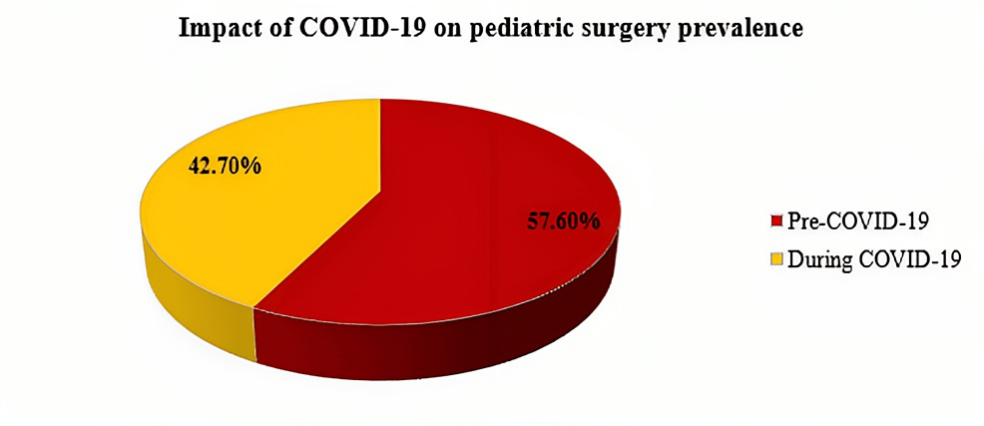
Impact of COVID-19 on pediatric surgery prevalence %: Percentage

Out of the patients who were admitted for surgical concerns (6086), the number of canceled surgeries before the COVID-19 pandemic was higher than the number during the COVID-19 pandemic (8.8% and 2.7%, respectively). Out of the patients who underwent surgeries (5704), it was indicated that the number of patients who underwent surgeries during the COVID-19 pandemic (42.7%) was less than those who underwent surgeries before the COVID-19 outbreak (57.6%). Full details are described in Table 2 and Figure 2.

**Table 2:** Impact of COVID-19 on pediatric surgery prevalence N: Number of cases

Category	Presentation Time	Total
Surgery performed (n=5704)	Pre-COVID-19	3288 (57.6)
	During COVID-19	2416 (42.7)

When comparing the effect of COVID-19 on the duration between the diagnosis and emergent surgical intervention, the period was significantly shortened during the COVID-19 pandemic with a median (IQR) of 3 (19.41) compared to before COVID-19 with a median (IQR) of 7 (13.25) (p-value = 0.037). See Table 3.

**Table 3:** Impact of COVID-19 on the diagnosis period of pediatric surgery IQR: Interquartile range.

Factors		Presentation Time		Total	p-value
	Category	Pre-COVID-19	During COVID-19		
Time from diagnosis to emergent surgical intervention	Median (IQR)	7 (13.25)	3 (19.41)	6 (15.05)	0.037

In Table 4, only 2% of the total patients had post-operation complications. No significant difference was seen in post-surgery complications, post-surgery PICU stay, outcome after surgery, or mortality rate before and during the COVID-19 era. Also, by comparing the impact of COVID-19 on post-operation details, the period of hospital stay was significantly shortened during the COVID period (p-value <0.001).

**Table 4:** Post-operation information IQR: Interquartile range

Factors		Presentation Time		Total	p-value
	Category	Pre-COVID-19	During COVID-19		
Post-surgery complications	Yes	63 (1.9)	52 (2.2)	115 (2)	0.529
	No	3226 (98.1)	2363 (97.8)	5588 (98)	
Post-surgery pediatric intensive care unit (PICU) length of stay	Median (IQR)	12 (65.3)	6 (19)	7 (19)	0.259
Outcome after surgery	Died	43 (1.3)	38 (1.6)	81 (1.4)	0.724
	Discharged to home improved	3244 (98.7)	2377 (98.4)	5621 (98.5)	
	Referred to another hospital	1 (0.03)	1 (0.03)	2 (0.03)	
Hospital stay	Median (IQR)	2 (3)	1 (2)	2 (3)	<0.001
Mortality rate after surgery	Yes	43 (1.3)	38 (1.6)	81 (1.4)	0.403
	No	3245 (98.7)	2378 (98.4)	5623 (98.6)	

**Figure 3:**
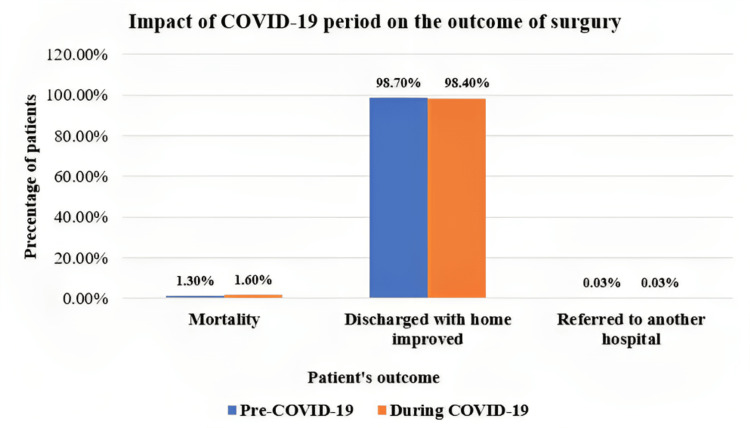
Impact of the COVID-19 period on the outcome of surgery %: Percentage

In Figure 3, patients who improved and were discharged home showed the highest percentage pre- and during COVID-19 (98.7% and 98.4%, respectively).

**Figure 4:**
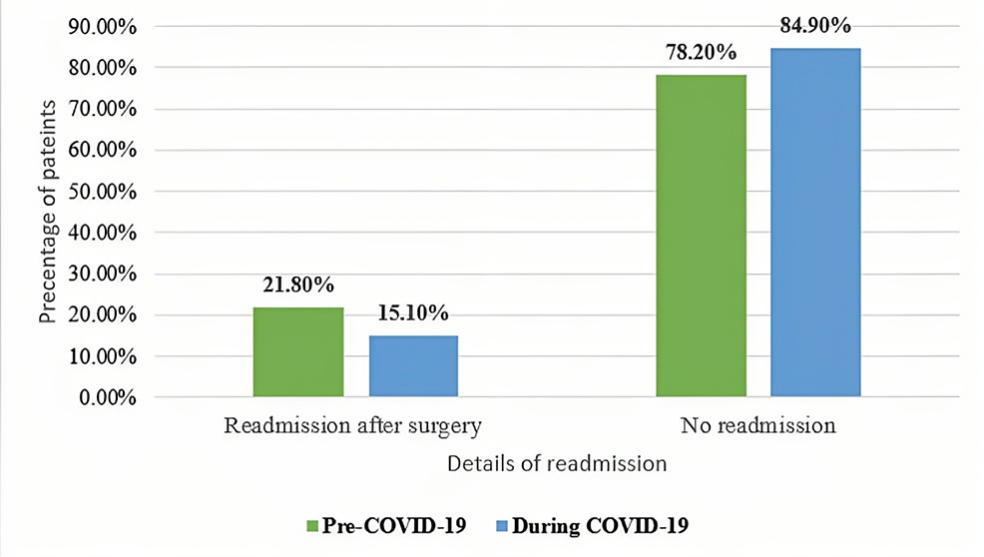
Impact of COVID-19 on the readmission rate %= Percentage

In Table 5 and Figure 4, details of readmitted patients are identified. About one-fifth of the patients were readmitted to the hospital (18.9%). Out of them, 35.7% performed another surgery. By comparing the impact of COVID-19 on readmission, the number of readmissions after surgery during COVID-19 (15.1%) was significantly decreased in comparison to the number of readmissions (21.8%) before COVID-19 (p-value <0.001).

**Table 5:** Patients’ readmission to the hospital after surgeries N: Number of cases

Factors		Presentation Time		Total	p-value
	Category	Pre-COVID-19	During COVID-19		
Readmission after hospital discharge (n=5704)	Yes	716 (21.8)	366 (15.1)	1082 (18.9)	<0.001
	No	2572 (78.2)	2050 (84.9)	4622 (81.1)	

## Discussion

The MCH in Al-Madina is the region’s main center for pediatric care. Regarding the general characteristics of pediatric surgery patients, male patients represent over 70%, likely due to the prevalence of conditions such as undescended testis, hydrocele, and testicular torsion in males. One study in Africa showed similar findings, where males represented 72% of the overall cases [12]. A large study in the United States revealed that males and females were almost equally represented at 50.9% and 49.1%, respectively [13]. However, our study in Al-Madina yielded different results, which may be attributed to the high number of circumcision surgeries in our population. It's worth noting that cultural differences play a significant role, as circumcision is a common practice for all male children in Muslim countries. Most surgeries performed before and after COVID-19 were on children aged 1-5. These results are in coherence with the results of a cross-sectional, cluster-based study that showed that the youngest children had the highest prevalence of surgical conditions [14]. This finding is important from the perspective of the healthcare system, as children in these age groups are susceptible to several non-surgical conditions as well. To ensure their good health, safe delivery of surgical services would be a prerequisite.

As evident by previous studies, the COVID-19 pandemic has led to a considerable decrease in the number of surgeries performed across all surgical specialties, encompassing both adult [15] and pediatric patients [9,16,17]. The results of this study indicate that there was a substantial decrease in the number of patients who underwent surgeries during the COVID-19 pandemic compared to the period before it. We found a reduction in both elective and emergency surgeries. Park et al. conducted a study in four middle-income countries in Africa and found a 32% decrease in surgical volume from March 2020 to April 2020 due to the pandemic. While surgical volume slowly recovered in the post-COVID period at a rate of one additional case per month, this rate was 20 cases per month lower than the pre-COVID trend [16]. Another study in China by Wei et al. revealed a significant reduction in the number of patients visiting hospitals and undergoing surgeries during the COVID-19 pandemic compared to the same period the previous year [9]. The study showed a decrease in the number of emergency cases and a statistically significant difference in the number of surgeries performed across different wards [9]. In Indonesia, Gunadi et al. found that the frequency of all elective surgeries during the pandemic was 1.5-4 folds lower compared to the three months before the outbreak. However, emergency pediatric surgical procedures remained consistent before and during the COVID-19 pandemic [17]. There are several possible reasons for the reduction in surgeries observed in this study.

The decrease in surgeries observed in this study could be due to various reasons. One of the factors could be implementing strict infection control measures in hospitals to prevent the spread of COVID-19. Another is the reduced capacity of hospitals to perform surgeries, as staff and equipment were diverted to COVID-19 patients. Additionally, all non-urgent elective surgeries were temporarily suspended to ensure sufficient hospital capacity to respond to the rapid spikes in COVID-19 cases and reduce the risk of COVID-19 transmission within hospitals [17]. Moreover, the relative lack of medical resources due to the increasing number of patients with COVID-19 and the accompanying economic downturn might also influence the management decisions for pediatric surgery patients [18]. Another possible factor could be the fear and anxiety amongst patients about visiting hospitals during the pandemic, leading them to postpone elective surgeries and non-urgent procedures. This could be due to concerns about exposure to the virus, the risk of contracting COVID-19, or disruptions caused by lockdown and quarantine measures [19]. Many studies support the conclusion that COVID-19 significantly influenced people’s surgical care-seeking behavior for pediatric surgery services and other medical and surgical services [9,11,20]. This research has also revealed that the overall reduction in elective and emergency surgeries did not impact the ratio of either type of surgery performed before and after the pandemic, with elective surgeries taking the majority of cases. These results contrast with a previous study that showed a decline in the proportion of elective surgical cases compared to emergency procedures [17]. It should be taken into consideration that our study spanned two years before and after the pandemic, in contrast to their study, which only covered three months before and after the outbreak. This may imply that the extended period for this research allowed for a better understanding of the pandemic and the necessary adjustments to be made, giving the healthcare system time to catch up on postponed elective surgeries. This led to no significant change in the ratio of elective to emergency surgeries.

The statistics show a noticeable decrease in canceled surgeries after the COVID-19 pandemic. This result could be due to the measures implemented to prevent and control the spread of the disease, which have reduced the overall number of elective surgeries performed. Before the pandemic, the cancellation rate was 8.8%, which is relatively high. Same-day surgery cancellations result in decreased operating room utilization, reduced productivity, and inconvenience for patients. Cancellations can be associated with various factors, including patient-related, surgeon-related, and hospital-related factors. Factors that affect patients include unforeseen illnesses, uncontrolled comorbidities, and an inability to comply with pre-operative instructions. As for surgeons, unexpected scheduling conflicts, insufficient operating room time or staff, or personal circumstances can all play a role. Hospital-related factors can include equipment, staff, resource shortages, and emergencies such as natural disasters. However, the cancellation rate dropped to 2.7% after the pandemic. This decrease can be attributed to several factors: the required pre-operative COVID test and stricter screening protocols helped to minimize same-day cancellations. Increased communication and education enhanced family engagement. Furthermore, improvements in communication channels between hospitals and patients due to the pandemic may have encouraged more careful patient selection and screening of patients for surgeries, resulting in fewer cancellations. The same results were observed in a previous study [21].

Regarding the significant changes in the demographic and clinical characteristics of the patients from pre- to post-COVID-19, a recent study showed there were more neonates and fewer adolescents who underwent surgery during the post-COVID period [16]. This is in coherence with the results of our study, as it shows a significant decline in the cases of pre-school age and school age but no significant affection in the cases of neonates and infants. This was likely due to neonates being present in the hospital following their births and neonatal care being prioritized in tertiary referral hospitals. For older children, the decrease in presentation may reflect a reduction in the rate of injuries [22] and a decrease in other viral infections due to lockdown and social distancing policies seen worldwide.

The COVID-19 pandemic has significantly impacted how emergent cases are managed. This study shows that the duration between diagnosis and surgical intervention for emergent cases was significantly shortened during the pandemic. A recent study on the adult general surgery population who presented to the ED found that during the pandemic, these patients experienced significantly reduced length of stay, waiting time, and time to hospital admission compared to the pre-pandemic baseline. The study findings correlate with ours, highlighting the possibility of more efficient hospital workflow and improved interdepartmental communication [23]. Conversely, a study conducted in Uganda regarding emergency surgical services revealed that patients were facing more delays in surgical care during the pandemic due to insufficient operating space and a shortage of surgeons. Moreover, they observed that the rise in COVID-19 cases was positively correlated with an increased proportion of care delays [24]. Multiple factors have contributed to decreased time between diagnosis and surgical intervention, including implementing new protocols and guidelines to limit virus exposure. The management of hospital beds has improved, and communication between admitting services and emergency providers has played a significant role in enhancing emergency care during the pandemic. Primary care clinics have been utilized to handle less complicated cases. Telehealth has been a great asset in allowing doctors to provide efficient and optimal care by reducing their workload.

The findings from Table 4 provide a detailed analysis of the impact of the COVID-19 pandemic on post-operative complications and other outcomes in pediatric surgical patients. The reassuringly low rate of post-operation complications indicates that the surgical teams involved in the care of these patients were competent and successful in their efforts to prevent complications during surgery and in post-surgical recovery. The lack of significant differences in post-surgery complications, duration of stay in the PICU, and mortality rates before and during the pandemic suggests that the healthcare system was able to maintain quality standards despite the added challenges posed by the pandemic. This is an important finding given the widespread concerns that the pandemic would result in lower-quality care and outcomes. In contrast to our results, the American College of Surgeons conducted a study that collected data from over 700 hospitals, revealing a rise in the rate of post-operative complications during the second quarter of 2020. This represented the highest proportion of operative complications since 2015, at approximately 13%. In the fourth quarter of 2020, they observed that the complication proportions had returned to the same levels as the first quarter of 2020 [25]. One possible reason for the variation in results between the studies is that our study lasted for two years before and after the pandemic, which allowed for better implementation of preventive measures. The shortened hospital stay period during the COVID-19 pandemic was an interesting finding and may reflect HCP's efforts to reduce the risk of COVID-19 transmission within hospitals. This shortened hospital stay was not observed among general surgery adult patients with emergency conditions, as they found that the length of stay was only significantly decreased in the ED but not in inpatient units [23].

The COVID-19 pandemic has also significantly impacted readmission rates in hospitals and healthcare facilities. This could be attributed to changes in patient care protocols, increased use of telemedicine, and improved infection control measures. In terms of readmission to the hospital, approximately one-fifth of the patients were readmitted. Out of them, a third were admitted to the surgical ward for additional surgery. The impact of COVID-19 is recognizable in the significant reduction in readmission rates. The decrease in readmission rates is consistent with the overall decline in admission rates, which was found to be considerable in our study and other studies [8,10,26].

Following the completion of this cross-sectional study on the impact of COVID-19 on pediatric surgery in Al-Madina, it was reported that one of the strengths of our study is the large sample size of 5,704 participants. This ensured a comprehensive representation of the population being studied. Furthermore, the extended period of observation allowed us to capture the effects of measures taken to address surgical challenges during the pandemic, thereby increasing the credibility of our findings. Although our study yielded valuable insights, it is important to acknowledge some limitations we encountered. Firstly, it was a retrospective study, which comes with inherent limitations. Additionally, during the observation period, there was a transition from paper to electronic systems, making data extraction challenging and potentially introducing biases. However, we took proactive measures to address this issue by carefully extracting data from hard-copy archives, which improved the accuracy of our results. Many areas need to be studied with regard to the impact of COVID-19 on pediatric surgery, such as how it has affected other regions and the effectiveness of alternative approaches to surgery during pandemics. It would also be beneficial to explore potential strategies to decrease the negative effects of the pandemic on the surgery field, like telemedicine or home-based care. Our study sheds light on how COVID-19 has impacted pediatric surgery in Al-Madina, and we hope it will guide future research and clinical practice in this field.

The fact that this is a single-center study is one of our key limitations. Nevertheless, considering that this was the only facility for specialist pediatric surgical care for most of the research period, we think we have a representative sample of newborns and early children who received surgical care.

## Conclusions

In conclusion, the COVID-19 pandemic has brought significant changes to the healthcare industry worldwide, and the impact on surgical cases has been a focus of interest for researchers. This study on the impact of COVID-19 on pediatric surgery cases in a referral hospital in the Al-Madina region of Saudi Arabia provides valuable insights into this matter. The study found that the pandemic has caused a considerable decline in the number of pediatric surgery cases compared to previous years. There was no significant change in the proportion of the types of surgeries performed before and after the COVID-19 outbreak. The longer observation period of this study likely allowed for a better understanding and adaptation to the pandemic situation. By understanding the impact of COVID-19 on pediatric surgical cases, healthcare professionals can develop strategies to mitigate potential long-term impacts on surgical services. Overall, this study contributes to strengthening our understanding of the pandemic's impact on healthcare services and can guide healthcare professionals in the effective management of pediatric surgical cases.
